# Assessment of inner and outer retinal layer metrics on the Cirrus HD-OCT Platform in normal eyes

**DOI:** 10.1371/journal.pone.0203324

**Published:** 2018-10-04

**Authors:** Sruthi Arepalli, Sunil K. Srivastava, Ming Hu, Peter M. Kaiser, Neeley Dukles, Jamie L. Reese, Justis P. Ehlers

**Affiliations:** 1 Cole Eye Institute, Cleveland Clinic, Cleveland, OH, United States of America; 2 Tony and Leona Campane Center for Excellence in Image-guided Surgery and Advanced Imaging Research, Cleveland Clinic, Cleveland OH, United States of America; 3 Department of Quantitative Health Sciences, Cleveland Clinic, Cleveland OH, United States of America; University of Florida, UNITED STATES

## Abstract

**Purpose:**

Ellipsoid zone (EZ) and outer retinal integrity are strongly linked to visual prognosis, but quantitative normative data is lacking. This study evaluates the EZ, outer retina, and inner retina in eyes without macular disease across a wide age spectrum.

**Methods:**

An IRB-approved study was performed for eyes without macular pathology undergoing Spectral Domain Optical Coherence Tomography (SD-OCT) scans on the Cirrus HD-OCT system (Carl Zeiss Meditec, Oberkochen, Germany). Scans were analyzed using a previously described automated EZ mapping tool with line-by-line manual verification. Segmentation included internal limiting membrane (ILM), outer nuclear layer/Henle fiber layer complex (ONL/HFL), EZ, and the retinal pigment epithelium (RPE). The output included metrics for the inner retina (ILM-OPL/HFL), outer retina (ONL/HFL-RPE), EZ-RPE area and volume, and *en face* EZ mapping. EZ-RPE attenuation on *en face* mapping was defined as EZ-RPE thickness < 20 um, and total attenuation was 0 um. Imaging parameters were assessed for the group and compared to age, sex, visual acuity and spherical equivalent.

**Results:**

167 eyes from 167 subjects were included. Mean age was 49.7 years (range 10–84 years). The mean foveal retinal thickness was 200.58 ± 19.22 um. Mean inner retinal thickness was 21.47 ± 13.60 um. Mean outer retinal thickness was 179.11 ± 18.52 um. Mean EZ-RPE thickness was 50.58 ± 6.01um. The mean EZ-RPE volume was 1.20 ± 0.10 mm^3^. Mean EZ attenuation percentage per macular map area was 0.87% ± 1.13% and mean percentage total attenuation was 0.12% ± 0.14%. Total and inner retinal thickness metrics decreased with age. Mean outer retinal thickness increased with age. EZ-RPE parameters were unchanged with age. However, EZ attenuation was negatively correlated with age.

**Conclusion:**

This study provides important information for inner and outer retinal parameters. Future research on quantitative EZ integrity can utilize this data for comparison.

## Introduction

Outer retinal changes are linked to multiple macular disease processes, as well as their prognosis and visual outcome. [1–19] Optical coherence tomography (OCT) provides a noninvasive, *in vivo*, and time efficient modality to reveal microscopic retinal and choroidal pathology, often not visible on clinical examination. [20–23] Previous studies have established normative quantitative metrics for total retinal thickness, individual layers and choroidal parameters using various methods. [24–39] However, few studies in particular have established a normative database for outer retinal and ellipsoid zone (EZ) metrics. [26, 28] This is particularly important given that the integrity of the outer retina and EZ have been directly linked to retinal function in numerous diseases, including age-related macular degeneration, achromatopsia, retinitis pigmentosa, ocular macular dystrophy, acute zonal occult outer retinopathy, hydroxychloroquine toxicity, ocriplasmin maculopathy, punctate inner choroidopathy, and following the repair of macular holes and retinal detachments. [1–8, 10, 11, 18, 19, 40]

The availability of an age-stratified normative dataset for inner and outer retinal parameters and metrics is important to provide comparative data for evaluation of pathologic alterations. This study aims to establish an outer retinal metrics dataset in eyes without macular pathology utilizing a recently developed, automated segmentation algorithm based on the internal limiting membrane, (ILM), outer nuclear layer/henle fiber layer complex (ONL/HFL), and various EZ parameters. This algorithm calculates various meters, including thickness, area, and volumetric measurements in eyes. [8]

## Methods

This was an IRB-approved, retrospective case series, which included 167 eyes of 167 subjects without macular disease. Inclusion criteria included Spectral Domain-OCT (SD-OCT) scanning with Cirrus HD-OCT system (Carl Zeiss Meditec, Oberkochen, Germany) between June 2011 and June 2016, availability of 512x128 macular cube with signal strength of 7 of 10 of greater, and sufficient OCT quality for analysis. The Cirrus HD-OCT platform recommends a signal strength of 6 or higher for quantitative analysis. [41] OCT had been obtained as part of standard of care for assessment of the fellow eye or due to suspected pathology in the study eye. Exclusion criteria for the study eye included intraocular surgery other than routine phacoemulsification, high myopia (i.e., ≥ 6.5 diopters), high hyperopia (i.e., ≥ 5.0 diopters), high amounts of astigmatism (i.e., > 4 diopters), any history of diabetic retinopathy, glaucoma, macular or neuro-ophthalmic disease, disc drusen, retinal/choroidal pathology on any line of the macular cube, and the presence of any retinal tissue architectural alteration on any line of the macular cube. Subject demographics and clinical data were gathered through the electronic medical record.

In the Cirrus Review software platform, each macular cube scan was reviewed line-by-line for the presence of any retinal/choroidal pathology and to confirm normal retinal anatomy and a lack of disruption of the retinal anatomy. Following confirmation of normal anatomy, the macular cube scans were exported for analysis.

The scans were imported into a previously described recently developed retinal layer analysis platform. [8, 42] In brief, this software platform enables linear, area and volumetric measurements of the various retinal layers through segmentation of the ILM, ONL/HFL, and EZ, as well as *en face* evaluation of the panmacular EZ-retinal pigment epithelium (RPE) thickness parameters. As documented in an earlier study, there is a high correlation between the automated measurements on repeat testing of the same eye. [8] The ONL/HFL was combined due to the challenges in discriminating HFL from the true ONL given the optical properties of the tissue and the lack of directional OCT information.

**Fig 1:**
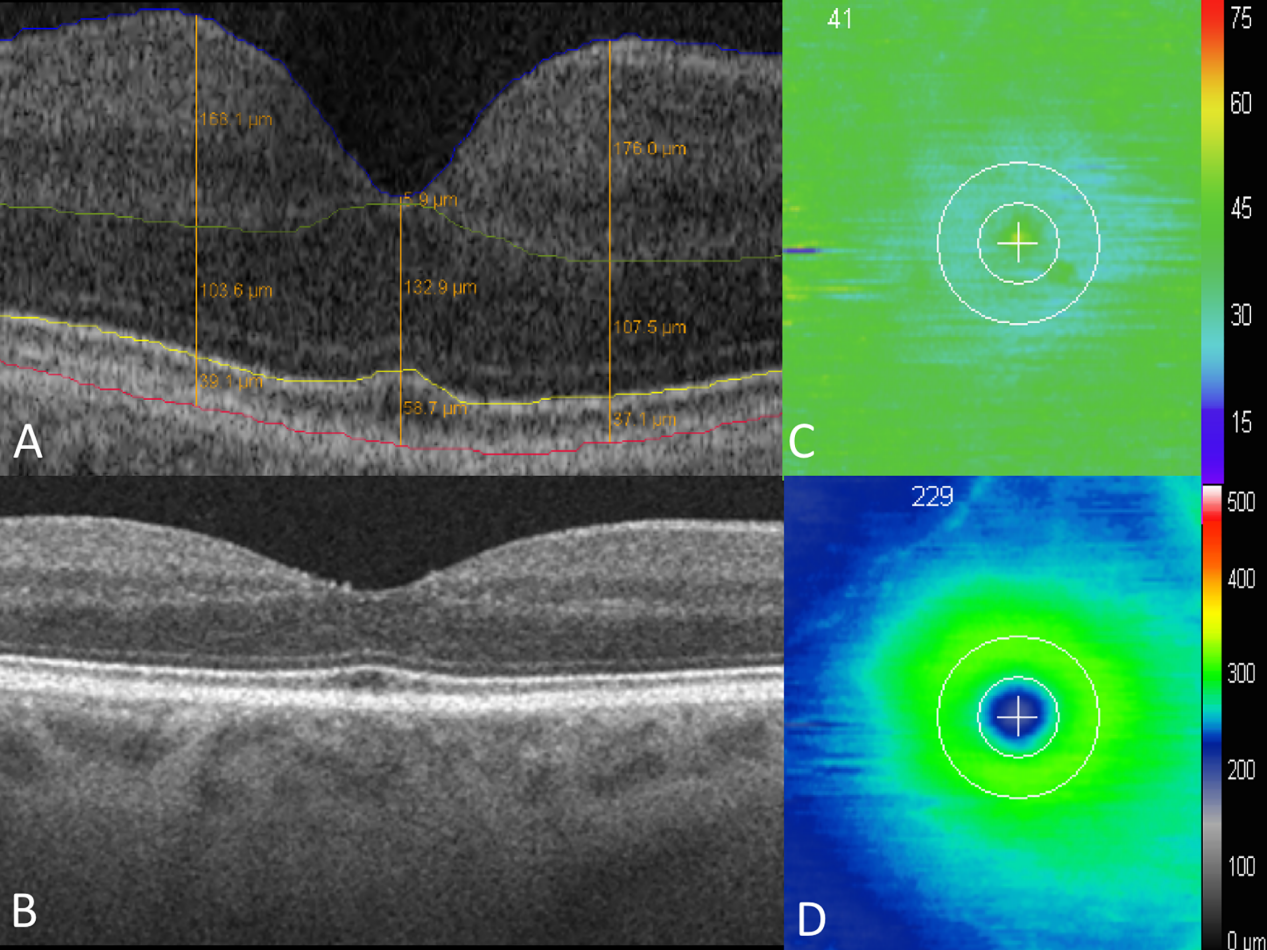
Segmentation map of macular OCT in a patient without macular pathology. (A) Segmentation of inner and outer retinal layers, at the inner limiting membrane (ILM, blue), outer limiting membrane (OLM, green), ellipsoid zone (EZ, yellow), and retinal pigment epithelium (RPE, red). (B) SD-OCT B scan. (C) EZ-RPE thickness map. (D) ILM-RPE thickness map.

A trained expert reader then reviewed each macular cube line-by-line for verification of appropriate segmentation. Any segmentation errors were manually corrected. Following initial segmentation validation, the segmented B-scans were reviewed by a single expert reader to assess for consistency of segmentation correction (N.D.). Once final segmentation validation was completed, multiple metrics were exported for analysis. Cross-sectional metrics included thickness, area, and volume measurements of layer parameters. Thickness measurements included inner retinal parameters from the ILM to the proximal boundary of the ONL. Outer retinal thickness was defined by the proximal ONL boundary to the RPE. EZ specific measurements were measured from the proximal end of the EZ to the proximal boundary of the RPE. Total retinal thickness measurements combined the measurements from the ILM to the RPE. Thickness measurements included central foveal thickness (CFT), juxtafoveal measurements on the foveal B-scan at 1 mm nasal and temporal to the fovea. (Fig 1)

Central foveal ONL/HFL-EZ area (which measured the total area occupied by the ONL/HFL to the EZ segments on a 6 mm horizontal B-scan) and volumetric measurements (which measured the total volume across the entire macular scan occupied by the space from the outer nuclear layer to the EZ) were also obtained. Similarly, central foveal EZ-RPE area, (which measured the total area occupied by the EZ and photoreceptor outer segments on a 6 mm horizontal B-scan) and volumetric measurements (which measured the total volume across the entire macular scan occupied by the space from the EZ to the RPE) were also obtained.

**Fig 2:**
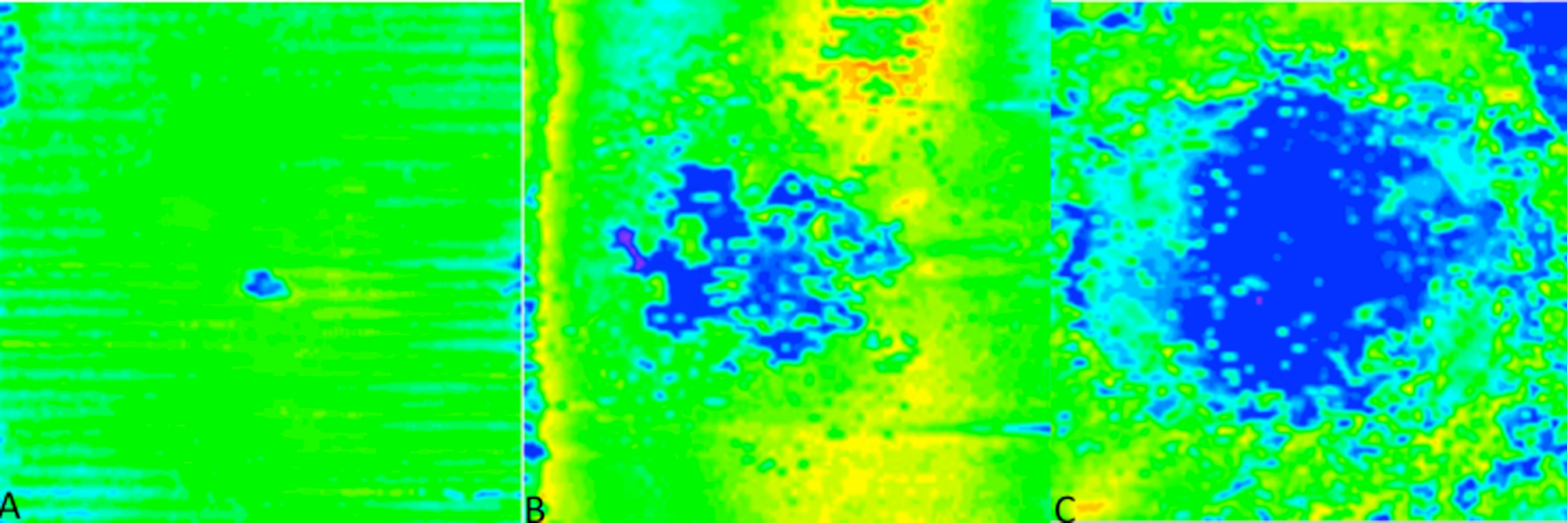
Comparison of EZ loss. (A) *En-face* map of the EZ layer with minimal loss, as shown by the majority of the green, from a patient included in this normative EZ study. (B) *En-face* map with moderate EZ loss, with more central blue, from a patient with Stargardt disease. (C) Severe loss, as shown by the expansive central blue areas, also from a patient with Stargardt disease.

*En face* mapping measurements related to EZ absence/total attenuation (e.g., EZ to RPE thickness = 0 microns) and EZ attenuation (EZ to RPE thickness < 20 microns), as previously described. These were represented as percentages of the overall macular cube.[8] (Fig 2) In the aforementioned study, over 99% patients without macular pathology had a thickness of 20 *u*m or more. These parameters were compared to age, visual acuity and spherical equivalent. These two factors were analyzed as an entire group (n = 167), and between males (n = 66) and females (n = 101). All analyzed metrics were analyzed by T test between the male and female cohort.

Patients were additionally divided into four cohorts based on age (1: 10–30, 2: 31–50, 3:51–70, 4:61–85), in order to analyze trends within segmentation layers. An ANOVA P test was performed to determine if there was a statistically significant difference between the quartiles.

In terms of statistical analysis, a Pearson’s product moment correlation coefficient (in short, R-value) was calculated between each OCT parameter versus age, visual acuity and spherical equivalent. We further applied T-test to evaluate the statistical significance of the R-value, in order to obtain the corresponding P-value. All statistical analysis was done in R version 3.4.3.

## Results

There were 167 eyes of 167 patients included in this study. The mean age was 49.7 years, (median, 55 years; range 10 to 84 years), and 101 (60.5%) of subjects were female and 66 (39.5%) were male. 146 (87.4%) eyes were phakic. (Table 1) Average visual acuity was 20/21 (range: 20/15 to 20/40). Average spherical equivalent was -0.48, (median, 0.0, range -5.63 to 4.3). Of the 167 patients, 4 had type 2 diabetes, and none had a current or previously documented history of diabetic retinopathy or macular edema. The reasons for obtaining OCT in the normal eye are documented in Table 1. All patients with a history of hydroxychloroquine use had normal visual field testing to confirm no toxicity. In patients with uveitis, a thorough history and examination had been performed to confirm unilateral involvement. Additional imaging was also obtained, as needed, to rule out bilateral disease.

**Table 1:** Assessment of inner and outer retinal layer metrics on the cirrus HD-OCT platform in normal eyes, demographic features.

Feature		All patients (n = 167)
Age (years) Mean (median, range)		49.7 years (55, 10–84 years)
Gender	Male	66 (39.5%)
	Female	101 (60.5%)
Spherical Equivalent (diopters) Mean (median, range)		-0.48 (0.0, -5.63 to 4.3)
BCVA	20/15	5 (7.5%)
	20/20	126 (71.6%)
	20/25	23 (13.0%)
	20/30	11 (6.3%)
	20/40	2 (1.2%)
Lens Status	Phakic	146 (87.4%)
	Pseudophakic	21 (12.6%)
Laterality	Right	87 (52.1%)
	Left	80 (47.9%)
Reason for OCT	Hydroxychloroquine use	39 (23.4%)
	Uveitis/endophthalmitis, contralateral eye	24 (12.2%)
	Baseline (initial visit)	19 (11.3%)
	Retinal vein occlusion, contralateral eye	17 (10.2%)
	CSR, contralateral eye	14 (8.4%)
	Macular hole, contralateral eye	14 (8.4%)
	Retinal detachment or retinal tear, contralateral eye	12 (7.2%)
	Cataract evaluation, contralateral eye	11 (8.4%)
	Macular edema, contralateral eye	9 (5.4%)
	ERM, contralateral eye	8 (4.8%)
	History of diabetes	4 (2.4%)
	AMD, contralateral eye	2 (1.2%)
	Coats disease, contralateral eye	1 (0.6%)

### Total retinal thickness parameters

#### Central foveal B-scan retinal thickness parameters

Overall mean central B-scan ILM-RPE thickness for the 167 eyes was 200.58 um (standard deviation: 19.22; range 158.4 to 264 um). In males, mean central ILM to RPE thickness was slightly higher than females.

#### Juxtafoveal nasal and temporal thickness parameters

Juxtafoveal nasal ILM to RPE thickness averaged 327.14 um (standard deviation: 19.04; range 267.8 to 397.1 um). The mean temporal juxtafoveal ILM to RPE thickness was lower, and measured 307.73 um (standard deviation: 17.85; range 250.9 to 353.9 um). When comparing the two groups, the juxtafoveal temporal ILM-RPE thickness was significantly lower in females versus males (p = 0.004). (Table 2)

**Table 2:** Assessment of inner and outer retinal layer metrics on the cirrus HD-OCT platform in normal eyes, metrics in entire cohort and sex breakdown.

		Total (N = 167) Average (standard deviation, range)	Males (N = 66) Average (standard deviation, range)	Females (N = 101) Average (standard deviation, range)	P values
Total Retina (ILM-RPE) (mean, standard deviation, range, um)	Central thickness	200.58 (19.22, 158.4 to 264)	204.06 (20.40, 164.2 to 264)	198.30 (17.78, 158.4 to 263.9)	0.07
	Juxtafoveal nasal thickness	327.14 (19.04, 267.8 to 397.1)	329.71(19.03, 285.4 to 365.7)	325.45 (18.95, 267.8 to 397.1)	0.156
	Juxtafoveal temporal thickness	307.73 (17.85, 250.9 to 353.9)	312.53 (16.55, 275.7 to 353.9)	304.59 (18.16, 250.9 to 346.1)	0.004
Inner Retina (ILM-OPL) (mean, standard deviation, range, um)	Central thickness	21.47 (13.60, 0 to 78.2)	23.52 (14.80, 0 to 78.2)	20.13 (12.67, 0.9 to 66.5)	0.12
	Juxtafoveal nasal thickness	201.56 (23.74, 138.8 to 287.7)	203.40 (22.09, 158.4 to 260)	200.36 (24.79, 138.8 to 287.7)	0.40
	Juxtafoveal temporal thickness	174.60 (18.81, 103.6 to 234.6)	179.62 (18.45, 138.8 to 234.6)	171.32 (18.40, 103.6 to 219)	0.005
Outer Retina (ONL/HFL-RPE) (mean, standard deviation, range, um)	Central thickness	179.11 (18.52, 125.2 to 228.8)	180.54 (18.13, 125.2 to 228.8)	178.17 (18.78, 129.1 to 217)	0.42
	Juxtafoveal nasal thickness	125.57 (18.43, 84to 166.2)	126.31 (18.97, 86 to 166.2)	125.09 (18.14, 84 to 164.2)	0.68
	Juxtafoveal temporal thickness	133.13 (16.26, 69.1 to 172.1)	132.91(16.46, 84.1 to 166.2)	133.27 (16.20, 69.1 to 172.1)	0.89
Ellipsoid zone (EZ-RPE) (mean, standard deviation, range, um)	Central thickness	50.58 (6.01, 37.1 to 68.4)	50.42 (6.25, 39.1 to 68.4)	50.68 (5.879, 37.1 to 66.5)	0.78
	Juxtafoveal nasal thickness	34.92 (5.52, 19.6 to 48.9)	31.10 (6.04, 19.6 to 48.9)	35.41 (5.13, 25.4 to 48.9)	0.17
	Juxtafoveal temporal thickness	33.39 (5.47, 21.5 to 58.7)	33.67 (5.63, 21.5 to 48.9)	33.20 (5.39, 21.5 to 58.7)	0.59
Area and Volumetric measurements	ONL-EZ Central Foveal Area (mm^2^)	0.55 (0.05, 0.43 to 0.73)	0.55 (0.06, 0.45 to 0.73)	0.55 (0.05, 0.43 to 0.64)	0.60
	ONL-EZ Volume (mm^3^)	2.87 (0.22, 2.41 to 3.57)	2.87 (0.24, 2.42 to 3.58)	2.88 (0.21, 2.41 to 3.36)	0.61
	EZ-RPE Central Foveal Area (mm^2^)	0.21 (0.02, 0.16 to 0.31)	0.21 (0.03, 0.16 to 0.31)	0.21 (0.02, 0.16 to .29)	0.88
	EZ-RPE Volume (mm^3^)	1.20 (0.10, 0.97 to 1.68)	1.2 (0.11, .97 to 1.68)	1.2 (0.1, .99 to 1.6)	0.76
	ONL-RPE Central Foveal Area (mm^2^)	0.76 (0.06, 0.63 to 0.96)	0.77 (0.06, 0.65 to 0.96)	0.76 (0.06, 0.62 to 0.92)	0.58
	ONL-RPE Volume (mm^3^)	4.08 (0.27, 3.53 to 4.84)	4.07 (0.27, 3.53 to 4.78)	4.08 (0.27, 3.57 to 4.84)	0.75
En face measurements	Twenty-micron EZ Map Coverage (%)	0.87 (1.13, 0.01 to 4.94)	0.99 (1.28, 0.12 to 4.93)	.78 (1.01, 0 to .62)	0.25
	Zero-micron EZ Map Coverage (%)	0.12 (0.14, 0 to 1.10)	0.1 (0.16, 0 to 1.1)	.13 (0.13, 0 to .62)	0.29

### Inner retinal thickness parameters

#### Central foveal inner retinal thickness parameters

The mean central ILM to OPL thickness for the 167 eyes was 21.47 um (standard deviation: 13.6; range 0 to 78.2 um). The male cohort had a higher central average thickness of in the female cohort, although not statistically significant. (Table 2)

#### Juxtafoveal nasal and temporal thickness parameters

Similarly to overall total retinal thickness trends, the juxtafoveal temporal ILM to OPL thickness measured less than the nasal measurements. As with the total retinal measurements, the juxtafoveal temporal inner retinal measurements were significantly lower in females than males (P = 0.005). (Table 2)

### Outer retinal thickness parameters

#### Central foveal B-scan outer retinal thickness parameters

The mean central ONL/HFL to RPE thickness for the 167 eyes was 179.11 um (standard deviation: 18.52; range 125.2 to 228.8 um). Males had a higher central ONL/HFL to RPE thickness as compared to females, but this was not statistically significant. (Table 2)

#### Juxtafoveal nasal and temporal thickness parameters

In contrast to the total retinal and inner retinal thickness parameters, juxtafoveal nasal ONL/HFL to RPE thickness was lower than juxtafoveal temporal ONL/HFL to RPE thickness. There were no significant differences between males and females.

#### Ellipsoid zone thickness parameters

In the total cohort, the central EZ to RPE thickness was 50.58 um (standard deviation 6.01; range 37.1 to 68.4 um). Males averaged 50.42 um for EZ to RPE thickness, and females averaged 50.68 um. There was no significant difference between males and females.

#### Juxtafoveal nasal and temporal thickness parameters

In the total group, juxtafoveal nasal EZ to RPE thickness averaged 34.92 um (standard deviation, 5.52; range 19.6 to 48.9 um), and temporal EZ to RPE thickness averaged 33.39 um (standard deviation, 5.47; range 21.5 to 58.7 um). In the males, temporal thickness higher, while in females, nasal thickness was higher. Neither of these was statistically significant. (Table 2)

### Outer retinal area metrics

Average ONL to EZ central foveal area measured 0.55 mm^2^ (standard deviation, 0.05, range, 0.43 to 0.73). Measurements were similar in males and females. (Table 2). EZ to RPE central fovea area was 0.21 mm^2^ (standard deviation, 0.02; range, 0.16 to 0.31 mm^2^). Measurements were similar with the male and female cohort as well. Overall, ONL to RPE central foveal area was 0.76 mm^2^ (standard deviation, 0.06, range, 0.63 to 0.96). There was no statistically significant difference between males and females.

### Outer retinal volume metrics

Mean ONL to EZ volume measured 2.87 mm^3^ (standard deviation, 0.22, range, 2.41 to 3.57), with similar values between males and females. Average EZ to RPE volume measured 1.2 mm^3^ (standard deviation, 0.1; range 0.97 to 1.68 mm^3^). Males and females measured similarly. In total, mean ONL to RPE volume measured 4.08 (standard deviation 0.27, range 3.53 to 4.84), with no statistically significant difference between male and female values for any of the volume measurements. (Table 2)

### En face mapping metrics of ellipsoid zone loss and attenuation

The average percentage of ellipsoid zone attenuation was 0.87% (standard deviation, 1.13%, range, .01% to 4.94%), and the average EZ total loss was .12% (standard deviation, 0.14%, range 0% to 1.1%). Males had slightly higher, but not statistically significant, average EZ attenuation. (Table 2) In terms of EZ loss, males and females had very similar averages. (Table 2). The most common areas for notable “attenuation” or “total attenuation” were areas of shadowing related to retinal blood vessels.

### Metrics in comparison to gender, age, best-corrected visual acuity and spherical equivalent

#### Gender

There was no significant difference between the two groups in terms of age, BCVA, or SE. The majority of thickness metrics were lower in the female cohort, while only two of these reached the level of significance: total retinal thickness and temporal inner retinal thickness. (Table 2)

#### Age

**Fig 3:**
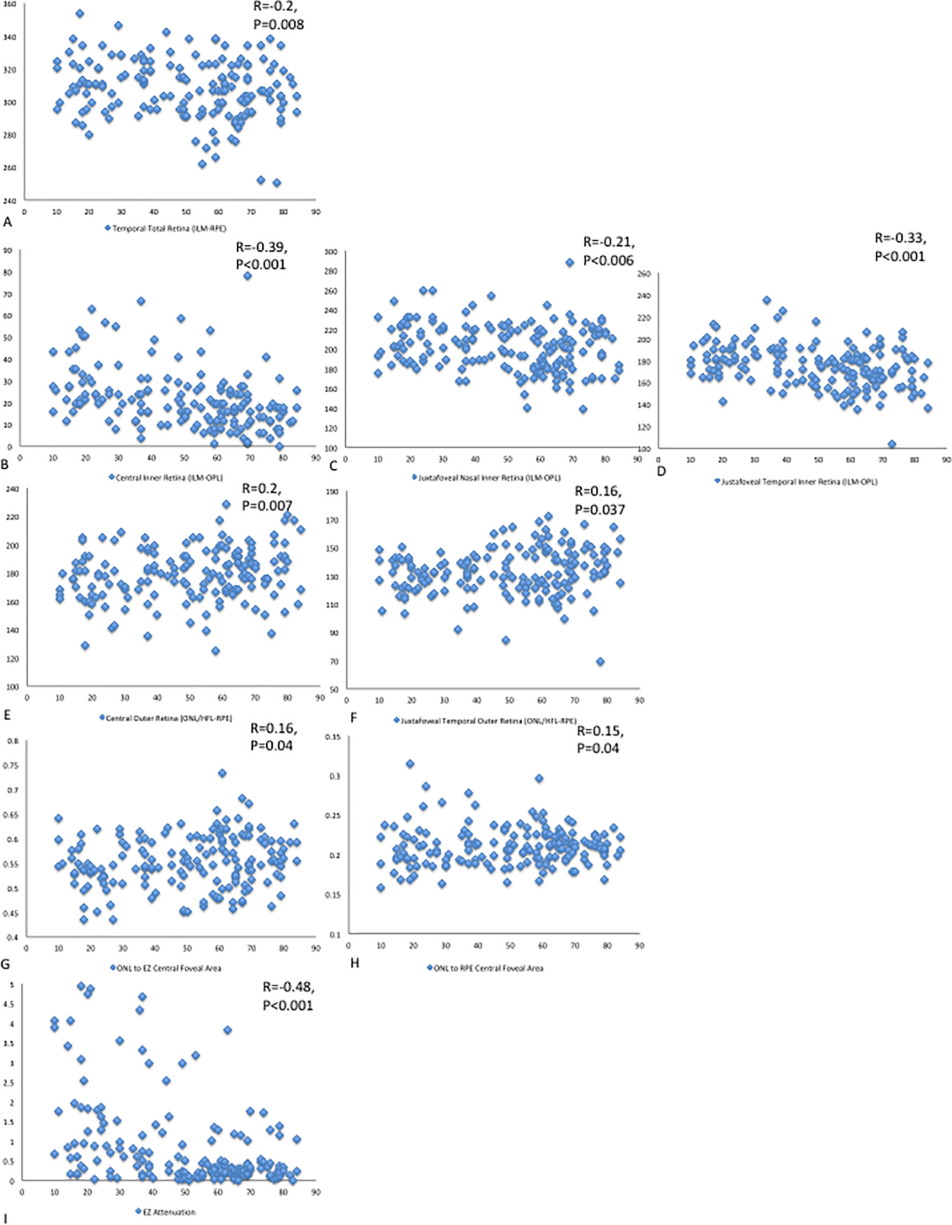
Statistically significant retinal parameters in comparison to age. (A) Temporal total retinal thickness showed a slightly negative correlation with age. (B) Central inner retinal thickness decreased moderately with age, while (C) nasal retinal thickness decreased slightly with age. (D) Similar to central retinal thickeness, temporal retinal thickness decreased moderately with age as well. (E) and (F) Central outer retinal thickness and temporal outer retinal thickness, respectively are both slightly negatively correlated with age. (G) and (H) ONL to EZ central foveal area and ONL to RPE central foveal area, respectively, both slightly increased with age. (I) EZ attenuation was strongly negatively correlated with age.

When comparing the entire cohort to age, several trends arise. Almost all of the total retinal measurements were negatively correlated to age in the total group and when stratified based on gender. In terms of total retinal thickness, temporal ILM to RPE thickness was statistically significantly but weakly negatively correlated with age, (r = -0.2; p = 0.008). In terms of the inner retina, the central, nasal and temporal measurements were also negatively correlated with age (r = -0.39, -0.21, -0.33; p<0.001, p = 0.006, p<0.001) respectively. For the outer retina, all measurements in all groups were weakly positively correlated with age. In terms of statistical significance, both central and temporal and central ONL/HFL to RPE thickness were weakly but positively correlated with age, (r = 0.2, r = 0.16; p = 0.007, p = 0.37, respectively). None of the ellipsoid zone thickness, area, volume or absolute loss measurements were significantly correlated with age. However, the amount of EZ attenuation was significantly negatively correlated with age (r = -0.48, p<0.001). (Table 3, Fig 3)

**Table 3:** Assessment of inner and outer retinal layer metrics on the cirrus HD-OCT platform in normal eyes. Comparison to age.

	Metric	All		Males (N = 66)			Females (N = 101)	
		R value	P value	R value		P value	R value	P value
Total Retina (ILM-RPE)	Central thickness	-0.07	.34	-0.14		.26	0.01	.94
	Juxtafoveal nasal thickness	-0.14	0.06	-0.19		0.12	-0.09	.34
	Juxtafoveal temporal thickness	-0.20	0.008	-0.29		0.02	-0.12	.22
Inner Retina (ILM-OPL)	Central thickness	-0.39	0.0000002	-0.33		0.0076	-0.43	<0.001
	Juxtafoveal nasal thickness	-0.21	0.006	-0.38		0.36	-0.09	0.36
	Juxtafoveal temporal thickness	-0.33	0.00001	-0.39		0.0001	-0.27	0.007
Outer Retina (ONL/HFL-RPE)	Central thickness	0.20	0.007	0.10		0.41	0.3	0.0024
	Juxtafoveal nasal thickness	0.12	0.12	0.25		0.043	0.03	0.79
	Juxtafoveal temporal thickness	0.16	0.037	0.16		0.21	0.17	0.09
Ellipsoid Zone	Central thickness	-0.06	.43	-0.209		0.09	0.05	0.60
	Juxtafoveal nasal thickness	0.10	0.19	0.04		0.76	.14	0.16
	Juxtafoveal temporal thickness	0.10	0.19	-0.012		0.91	0.20	0.044
Area and Volume measurements	ONL to EZ central foveal area	0.16	0.04	0.23		0.06	0.1	0.3
	ONL to EZ volume	0.09	0.25	0.16		0.20	0.02	0.81
	EZ to RPE central foveal area	0.03	0.64	-0.167		0.18	0.21	0.03
	EZ to RPE volume	0.05	.52	-0.09		0.46	0.17	0.07
	ONL to RPE central foveal area	0.15	0.04	0.15		0.23	0.17	.08
	ONL to RPE volume	0.09	0.23	0.10		0.40	0.08	0.40
En face Ellipsoid Zone mapping	Attenuation (<20 um)	-0.48	0.000000001	-0.45		0.00013	-0.43	<0.001
	Loss (0 micron)	0.06	.43	-0.001		0.99	0.10	0.31

Additionally, the total cohort was divided into quartiles based on age. (Table 4) On the whole, total retinal thickness (ILM-RPE), along with temporal and nasal thickness, tended to measure slightly higher in younger quartiles than older quartiles, with a significant difference between quartiles when comparing temporal total thickness. (P = 0.03) The inner retinal thickness was also found to be higher in younger quartiles than older quartiles, with a significant P value when examining central, temporal and nasal thicknesses. (P<0.001, P = 0.003, P<0.001) Outer retinal thickness trended to increase from quartile 1 to quartile 4. There was no statistical difference between the four quartiles. There was no clear relationship between any of the area of volumetric measurements in the outer retina and quartiles. Moreover, percentage of EZ attenuation tended to be higher in quartile 1 and 2, and over 50% decreased in quartile 3 and 4. This difference was very statistically significant (P<0.001). (Table 4).

**Table 4:** Assessment of inner and outer retinal layer metrics on the cirrus HD-OCT platform in normal eyes. Comparison across quartiles.

	Metric	Quartile 1 (Age 10–30) Average (standard deviation, range)	Quartile 2 (Age 31–50) Average (standard deviation, range)	Quartile 3 (Age 51–70) Average (standard deviation, range)	Quartile 4 (Age 71–85) Average (standard deviation, range)	P value
Total Retina (ILM-RPE)	Central thickness	203.38 (20.57, 166.2 to 263.9)	201.65 (16.23, 158.4 to 230.7)	199.16 (19.53, 161.2 to 264)	198.156 (20.57, 164.2 to 236.5)	0.63
	Juxtafoveal nasal thickness	330.83 (19.3, 285.4 to 373.5)	330.89 (14.93, 304.9 to 365.7)	324.25 (20.88, 267.8 to 397.1)	323.2 (17.78, 275.6 to 350)	0.14
	Juxtafoveal temporal thickness	312.04 (16.78, 279.6 to 353.9)	312.14 (13.96, 291.3 to 342.1)	303.50 (18.28, 261.9 to 383.3)	305.3 (21.1, 250.9 to 338.2)	0.03
Inner Retina (ILM-OPL)	Central thickness	29.38 (13.5, 7.8 to 62.6)	23.79 (13.99, 3.9 to 66.5)	17.9 (12.44, 0.9 to 78.2)	14.5 (8.82, 0 to 41.1)	<0.001
	Juxtafoveal nasal thickness	210.23 (21.52, 176 to 260)	206.64 (20.25, 168.1 to 254.2)	194.24 (24.59, 140.8 to 287.7)	199.1 (24.4, 138.8 to 230.7)	0.003
	Juxtafoveal temporal thickness	182.58 (14.84, 142.7 to 213.1)	180.40 (21.09, 148.3 to 234.6)	168.97 (15.77, 134.9 to 205.3)	167.81 (21.81, 103.6 to 205.3)	<0.001
Outer Retina (ONL/HFL-RPE)	Central thickness	174 (17.9, 129.1 to 209.2)	177.86 (16.64, 134.9 to 205.3)	181.28 (18.02, 125.2 to 228.8)	183.65 (22.08, 136.9 to 220.9)	0.13
	Juxtafoveal nasal thickness	120.6 (19.47, 84 to 166.2)	124.25 (17.47, 88 to 154.4)	130.01 (17.7, 86 to 164.2)	124.10 (18.33, 86 to 160.3)	0.07
	Juxtafoveal temporal thickness	129.46 (11.38, 103.6 to 150.5)	131.73 (16.63, 84.1 to 162.2)	134.53 (16.95, 99.7 to 172.1)	137.50 (19.78, 69.1 to 166.2)	0.2
Ellipsoid Zone	Central thickness	50.22 (6.44, 41.1 to 62.6)	52.46 (6.26, 43 to 68.4)	50.38 (5.89, 37.1 to 66.5)	49.0 (4.75, 39.1 to 58.7)	0.14
	Juxtafoveal nasal thickness	33.80 (6.63, 19.6 to 48.9)	35.29 (5.10, 25.4 to 46.9)	35.48 (5.02, 27.4 to 46.9)	34.8 (5.4, 25.4 to 48.9)	0.48
	Juxtafoveal temporal thickness	32.32 (5.52, 23.5 to 48.9)	33.61 (6.0, 21.5 to 48.9)	33.89 (5.57, 21.5 to 58.7)	33.54 (4.32, 25.4 to 43)	0.54
Area and Volume measurements	ONL to EZ central foveal area	0.54 (0.05, 0.43 to 0.63)	0.54 (0.04, 0.45 to 0.63)	0.56 (0.06, 0.455 to 0.73)	0.56 (0.04, 0.46 to 0.63)	0.10
	ONL to EZ volume	2.84 (0.2, 2.42 to 3.36)	2.86 (0.20, 2.45 to 26)	2.89 (0.27 (2.4 to 3.58)	2.91 (0.16, 2.54 to 3.17)	0.55
	EZ to RPE central foveal area	0.20 (0.03, 0.16 to 0.31)	0.21 (0.02, 0.16 to 0.28)	0.21 (0.02, 0.16 to 0.3)	0.21 (0.01, 0.17 to 0.23)	0.70
	EZ to RPE volume	1.20 (0.14, 1.0 to 1.68)	1.19 (0.09, 1.04 to 1.38)	1.21 (0.10, 0.98 to 1.6)	1.19 (0.07, 0.96 to 1.31)	0.65
	ONL to RPE central foveal area	0.75 (0.05, 0.62 to 0.87)	0.75 (0.05, 0.6 to 0.86)	0.77 (0.06, 0.65 to 0.96)	0.77 (0.04, 0.67 to 0.84)	0.09
	ONL to RPE volume	4.04 (0.25, 3.6 to 4.84)	4.05 (0.26, 3.6 to 4.65)	4.10 (0.31, 3.53 to 4.8)	4.11 (0.19, 3.66 to 4.40)	0.55
En face Ellipsoid Zone mapping	Attenuation (20 um)	1.58 (1.44, 0.04 to 4.93)	1.08 (1.29, 0.018 to 4.67)	0.44 (0.65, 0.01 to 3.8)	0.47 (0.47, 0.01 to 1.71)	<0.001
	Loss (0 um)	0.10 (0.11, 0 to 0.42)	0.13 (0.21, 0 to 28)	0.12 (0.12, 0 to 0.62)	0.13 (0.12, 0 to 0.41)	0.75

#### Visual acuity

When compared against VA, none of the metrics in the general cohort, male or female cohorts were significantly correlated.

#### Spherical equivalent

When comparing SE to the various metrics, the nasal ONL/HFL to RPE and central ONL/HFL to RPE were weakly positively correlated with spherical equivalent (r = 0.16, p = 0.03; and r = 0.25, P<0.001, respectively). These correlations also existed in the female cohort.

## Discussion

Pathological changes in the retina, and in particular, the outer retina and ellipsoid zone are linked to visual decline. [3, 6, 7] Comparing these changes to age is instrumental in delineating pathology from normal aging processes. The segmentation methods described in this paper have been validated in previous studies, and provide an accurate, reproducible measurement tool for various retinal metrics. [8] While previous publications have published on total retinal thickness and inner retina thickness, few have published on outer retinal metrics. [24, 26]

The measurements for total retinal thickness with this automated segmentation technique closely mirror previous studies. [27, 31] Bressler et al, using status OCT, found an average CST of 209 um in males and 194 um in females; this is very close to our measurements of 204.06 um and 198.3 um, in males and females, respectively. [31] Moreover, they found no significant difference in measurements between non-diabetics and diabetic patients with no retinopathy. All four of the diabetic patients included in our study had no retinopathy ever recorded. Similarly, Kashani et al published on average male and female foveal thickness measuring 201.8 um and 186.9 um, respectively. Overall, we found that the total retinal thickness and inner retinal layers, defined as from the ILM to the outer boundary of the OPL, almost always had a negative correlation with age, with a stronger relationship existing when examining the inner retina alone. This correlation between age and total retinal thickness and inner retinal thickness has been reported previously. [26, 28, 38, 43, 44] Reports have highlighted, in particular, the decline of the retinal nerve fiber layer contributing to the decrease in total retinal thickness. [38, 44, 45] The trend towards thicker nasal juxtafoveal measurements reflects the thicker nerve fiber layer anatomically, and has been reflected in the literature as well. [26]

Outer retinal thickness, measured by our segmentation techniques of the outer nuclear layer to the RPE, always increased with age, albeit with a weak correlation factor in certain metrics. Tong et al examined the ONL/HFL layer in particular with directional OCT and also found a general trend towards thicker measurements with increased age. [24] Interestingly, Ooto et al concluded that the outer fovea does not decrease with age, as opposed to their inner retinal measurements, which generally did show a negative relationship to age. [26] One important difference to note is that the OPL and ONL/HFL measurements were combined in that report. However, in concordance with both these works, we found a general positive correlation with the outer retinal layers and age, instead of a decrease with age as we found in the inner retinal layers.

In particular, the EZ has garnered attention as a possible surrogate for visual acuity or retinal function.[1–4, 6–8, 10–19, 40, 46] Currently, the EZ, present as the second to last hyperreflective band on OCT, is thought to correspond to the ellipsoid portion of the inner segments.[22, 23] Establishing a normal eye dataset for these metrics is imperative to compare to future trials for interventional medications and to monitor disease progression, such as in conditions that primarily effect the EZ (e.g., ocriplasmin maculopathy, hydroxychloroquine retinopathy, macular telangiectasia.) [9, 47] Many studies in the past have focused on binary assessment: assessing for presence or disruption. [10, 40] Some studies have attempted to evaluate EZ reflectivity or compare reflectivity to the ELM, but failed to establish quantifiable parameters for the expected ellipsoid values.[1–4] Birch et al and Hood et al established outer segment thickness values in a small groups of patients, but did not correlate these with age. [13, 14, 19] Our study provides an in-depth assessment of outer retinal metrics across a large study group and age-range. Not only does this study provide linear measurements, but utilizing a novel software platform this report provides volumetric and *en face* assessments.

There has been no clear consensus in the literature on the normal ellipsoid zone changes associated with aging. [48–50] One report examines the anatomical development of the human fovea, and details how the foveal cone diameter decreases markedly after birth, with development of the cone outer and basal axon processes up to at least four year of age. [48] The authors conclude that outer segment length and cone packing density are still approximately half of that as adults, even at age four, meaning that younger adults may have shorter cone outer segments. These factors may play into the slight correlation between increasing thickness in our patients with age. In another report, the normal human retina is histologically examined in various decades (from the 2nd to the 9th), and found that the foveal cone density had a surprisingly large range, with a variation of 25–40% in cone density per each age group. They also did not find a relationship between age advancement and foveal cone decline, suggesting foveal cone density does not significantly decline with age. Even more interestingly, they found a similar cone foveal density in a donor at age 95 to a 19 year old patient.[50]

Overall in our study, the EZ measurements were very similar between the two groups, without significant difference between males and females. Additionally, we found that no definite trend in ellipsoid zone thickness when compared to age. These small differences may from the previously described expected high variability.

Similarly, we found that the overall parameters for area and volume were homogenous across the age group and between males and females. We did find a significant negative correlation between age and percentage of EZ attenuation. This may be from poorer cooperation in younger patients resulting in less reliable scans (e.g., artifact). More likely, this may be due to other anatomical factors, such as increased vessel shadowing in younger patients due to larger vessel caliber, causing perceived EZ attenuation by the software. [51, 52] It could also be related to previously mentioned delayed development of cone outer segments as compared to adults. However, overall the percentage remained quite small of attenuation or total attenuation.

We also found that males tended to have higher thickness measurements as compared to females in terms of total retinal thickness, inner retinal thickness and outer retinal thickness. A trend towards higher measurements in males has been reflected in many previous works. [31, 33, 39, 45] We did not see a trend towards thicker measurements in males when examining the ellipsoid zone metrics; this may be from relatively small micron changes between the two groups.

This work is not without its limitations. Previous works have commented on a racial impact on retinal layer thickness; in particular, African Americans tend to have thinner measurements than Caucasian counterparts. [27, 29, 32] As this was a retrospective study racial information was unavailable for analysis. Furthermore, the analysis based on spherical equivalent is difficult to extrapolate, as spherical equivalents were used from the date of OCT, which may not accurately depict their original spherical equivalent, if they had cataract surgery. Additionally, the wide range of refractive error undoubtedly impacts the magnification error, and Cirrus software does not take into account this magnification. Various other factors, including head tilt when taking the OCT, and measuring exact fixation were not accounted for, which may skew the results. These results can only be extrapolated to the Cirrus software as well, as this was used for the data collection. Moreover, various systemic conditions, such as heart disease and hypertension have been linked to retinal thickness parameters, but these medical records were not available for every patient, limiting our ability to assess their impact on the retina.

The results of this work may be able to serve as a benchmark for comparative assessment in outer retinal evaluation of pathologic conditions. [42] The lack of clear relationship between age and ellipsoid parameters supports previously published data and removes a possible confounding factor in later analyses examining large age ranges. This extensive dataset may serve as an important comparative cohort for future studies in various disease conditions impacting the outer retina.

## Supporting information

S1 File Data set for retinal measurements. (XLSX)
